# Identification of serum protein biomarkers for utrophin based DMD therapy

**DOI:** 10.1038/srep43697

**Published:** 2017-03-02

**Authors:** Simon Guiraud, Benjamin Edwards, Sarah E. Squire, Arran Babbs, Nandini Shah, Adam Berg, Huijia Chen, Kay E. Davies

**Affiliations:** 1 Medical Research Council Functional Genomics Unit at the University of Oxford, Department of Physiology, Anatomy and Genetics, Oxford, OX1 3PT, United Kingdom

## Abstract

Despite promising therapeutic avenues, there is currently no effective treatment for Duchenne muscular dystrophy (DMD), a lethal monogenic disorder caused by the loss of the large cytoskeletal protein, dystrophin. A highly promising approach to therapy, applicable to all DMD patients irrespective to their genetic defect, is to modulate utrophin, a functional paralogue of dystrophin, able to compensate for the primary defects of DMD restoring sarcolemmal stability. One of the major difficulties in assessing the effectiveness of therapeutic strategies is to define appropriate outcome measures. In the present study, we utilised an aptamer based proteomics approach to profile 1,310 proteins in plasma of wild-type, *mdx* and Fiona (*mdx* overexpressing utrophin) mice. Comparison of the C57 and *mdx* sera revealed 83 proteins with statistically significant >2 fold changes in dystrophic serum abundance. A large majority of previously described biomarkers (ANP32B, THBS4, CAMK2A/B/D, CYCS, CAPNI) were normalised towards wild-type levels in Fiona animals. This work also identified potential *mdx* markers specific to increased utrophin (DUS3, TPI1) and highlights novel *mdx* biomarkers (GITR, MYBPC1, HSP60, SIRT2, SMAD3, CNTN1). We define a panel of putative protein *mdx* biomarkers to evaluate utrophin based strategies which may help to accelerate their translation to the clinic.

Duchenne muscular dystrophy (DMD) is a lethal X-linked recessive disorder caused by mutations in the dystrophin gene^1^. This disorder affects 1 in 5000 boys^2^ and is characterized by a progressive muscle wasting leading to loss of ambulation by 8–12 years of age^3^ and death by early adulthood due to cardiorespiratory failure^4^. Dystrophin, an essential link between the dystrophin associated protein complex (DAPC) at the sarcolemma and the cytoskeleton, maintains the strength, flexibility and stability in skeletal muscles^5^. In the absence of dystrophin, the myofibres are more susceptible to contraction-induced injury which results in muscle wasting and premature death^6^.

There is currently no effective treatment for the disease. Glucocorticoid treatment is the current standard of care which delays the loss of ambulation by 3–4 years^7^,^8^ but shows no long treatment benefit and is often associated with debilitating side effects^9^,^10^,^11^. The urgency to seek a therapy for DMD has resulted in parallel efforts to develop exon skipping^12^,^13^, termination codon read through^14^, dystrophin gene replacement or editing therapies^15^,^16^ and non-dystrophin strategies^17^,^18^,^19^ such as utrophin modulation^20^,^21^. However, despite the recent accelerated approval of Exondys 51 (eteplirsen) in US, disappointing clinical trials results^22^ and failure of approval from the FDA for Ataluren^23^ and Kyndrisa^24^ drugs rekindle discussions about clinical trials designs and endpoints.

We have focused on utrophin modulation because it is applicable to all DMD patients irrespective of their dystrophin mutation. Utrophin is found at the sarcolemma *in utero* and is progressively replaced by dystrophin during development^25^,^26^,^27^. In adult skeletal muscles, utrophin is expressed and enriched at the neuromuscular and myotendinous junctions^28^ and found at the sarcolemma in regenerating myofibres^29^. Despite subtle differences, utrophin shares 80% of homology^30^ with the dystrophin protein and has functional redundancy^31^,^32^,^33^,^34^. Utrophin is increased 1.8 fold in the mouse mdx model of the disease due mainly to regenerating fibres. Using transgenic *mdx* mice expressing high levels of utrophin (Fiona), we have demonstrated that increasing utrophin expression 3–4 fold prevents the development of pathology^35^,^36^. In partnership with Summit Therapeutics, we have developed small molecules which increase the levels of utrophin and prevent pathology in the *mdx* mouse model^21^,^37^. One of these, Ezutromid (formerly known as SMT C1100) has progressed into clinical development. Ezutromid has an excellent safety profile^20^,^38^, and recently entered into phase 2 trial^39^. We have reported a second generation compound, chemically related to Ezutromid, with improved physicochemical properties and a robust metabolism profile which ameliorates sarcolemmal stability and prevents the pathology through a significant reduction of regeneration, necrosis and fibrosis and provides functional enhancement^21^. These data emphasize the potential of utrophin modulation as a disease-modifying therapeutic strategy for all DMD patients.

Current clinical trials have used the analysis of the restoration of dystrophin as a biomarker. However this relies on invasive muscle biopsies which only provide semi-quantitative measures due to the small size of the tissue sample. The utility of the quantification of dystrophin as a biomarker is still under debate and limited by current western blot and immunofluorescence microscopy methodologies^40^. Furthermore, therapeutic strategies deliver different efficacy depending on the muscle type. In consequence, the correlation between the dystrophin level in a biopsy of one muscle type and the overall clinical improvement is under question. Currently, most clinical trials for DMD rely on standardized physical assessments such as the 6 minute walk distance test (6MWDT)^41^, the North Star Ambulatory Assessment (NSAA)^42^ as well as quantitative muscle strength tests^43^,^44^. These physical tests are useful readouts for determining the whether a treatment slows disease progression but these endpoints are limited to ambulatory patients only, often challenging to implement specially in young patients and suffer from high inter-patient variability due to the variable natural history of the disease.

Recently, less invasive approaches to monitor disease progression and response to treatment in DMD patients have emerged with Magnetic resonance imaging and T2 mapping^45^,^46^. While these approaches are useful in monitoring muscle loss, fat and progression of the disease as cardiac function, they do not provide a direct muscle function read-out, are laborious and subject to a number of limitations such as high cost and low through-put.

Thus there is an urgent need for minimally invasive biomarkers^47^,^48^, which can be used as outcome measures in pre-clinical studies and clinical trials for DMD therapeutics. Blood fluid samples are simple to obtain and provide insight into the circulating protein in the entire body in normal as pathological conditions^49^. Initially, serological level of creatine kinase (CK) was used to screen for DMD in newborns^50^,^51^. However whereas serum muscle CK reflects sarcolemma damage and is a useful tool for diagnosis, this marker is not suitable for monitoring the extent of the pathology, disease progression and response to therapy as it is influenced by age, exercise and stress^52^. In addition, serum CK may also be elevated in asymptomatic individuals. Over the last 5 years, work profiling different types of molecular biomarkers including miRNA, protein and metabolites in serum, plasma and/or urine of DMD patients have been reported^53^,^54^,^55^. Circulating protein markers such as carbonic anhydrase III (CA-III), myoglobin, TIMP-1^55^,^56^, MMP9^57^ or MYOM-3^58^, which are simple to integrate into clinical workflows are emerging as valuable and useful biomarkers.

Current methodologies such as two-dimensional gel electrophoresis are limited to highly expressed proteins. Mass spectrometry is challenging to analyse with serum/plasma samples due to high dynamic range in abundance of different proteins as albumin, fibrinogen, immunoglobulins and macroglobulin which represent 90% of the circulating proteome. This can mask any less abundant potential biomarkers. To overcome these limitations, sensitive high throughput “omic” platforms such as affinity proteomics approach were recently introduced. This multiplexing methodology using 384 antibodies directed against 315 different proteins identified new protein biomarker as Troponin T, fast skeletal muscle (TNNT3), myosin light chain-3 (MYL3) and plastin-2 (LCP1) in a total of 190 blood samples from DMD patients^59^. Another serum proteome profiling study using a combination of high precision mass spectrometry and stable isotope labelling in mammals (SILAM) strategy quantify levels of 1,500 proteins in 15 DMD sera and two independent *mdx* model for DMD and successfully identified 20 protein biomarkers^55^. More recently, a new aptamer-based affinity purification approach (SOMAscan) was developed^60^ and successfully used to query 1,129 proteins in serum samples from 93 DMD patients^56^. Forty-four were identified at a False Discovery Rate of 1.0% to be significantly increased (24 proteins) or decreased (20 proteins) in DMD patients *vs.* controls. The SOMAscan technology was also used with *mdx* mice to define novel protein biomarkers^61^. These recent highly sensitive high throughput technologies provide a comprehensive panel of serum protein biomarkers which should be valuable tool to monitor treatment efficacy in future pre-clinical and clinical studies.

In this study, we carried out comprehensive serum proteome profiling using the 1,310-plex SomaScan assay in wild-type, *mdx* and Fiona mice to define panels of serum protein DMD markers applicable to utrophin modulation based therapies. The definition of therapeutic monitoring serum biomarkers in different utrophin level contexts should accelerate the development of small oral utrophin drugs for a quicker translation to DMD patients.

## Results

### Serum protein biomarkers profiling in *mdx*

In order to define robust circulating biomarkers for utrophin based strategies, we collected serum from 7 week old C57, *mdx* and utrophin transgenic *mdx* Fiona mice (n = 7). In dystrophic tibialis anterior (TA) muscle, mainly due to regeneration^29^, utrophin is increased by 2 fold in *mdx* compared to C57 animals and inconsistently localised at the muscle membrane of small regenerating fibres (Fig. S1A,C). In the transgenic *mdx* Fiona mouse, utrophin is expressed at a high level (4 fold compared to *mdx*) and uniformly distributed at the sarcolemma (Fig. S1A,B). Consequently, muscle membrane stability is improved, regeneration and necrosis are reduced to wild-type levels and muscle function is fully restored (Fig. S1C–E).

In this study, we used the SOMAscan platform to profile serum abundance of 1,310 SOMAmers (Slow Off-rate Modified Aptamers) in C57, *mdx* and Fiona samples (n = 7). These complexes, able to recognize with high specificity and sensitivity specific conformational epitopes of proteins, were precipitated and protein concentrations quantified on an Agilent hybridisation chip^60^. SOMAscan methodologies used three dilutions (0.5%, 2% and 5%) per sample to increase the dynamic range of detection and a unique set of SOMAmer reagents were assigned to one of three dilution sets. Quality controls showed that probe hybridization was in the acceptable range 0.4–2.5 for all the samples (Fig. S2A,B) and that median normalization scale factor was similar between groups of animals (Fig. S2A,C). All samples successfully passed quality control checks and were included in subsequent analyses.

**Figure 1:**
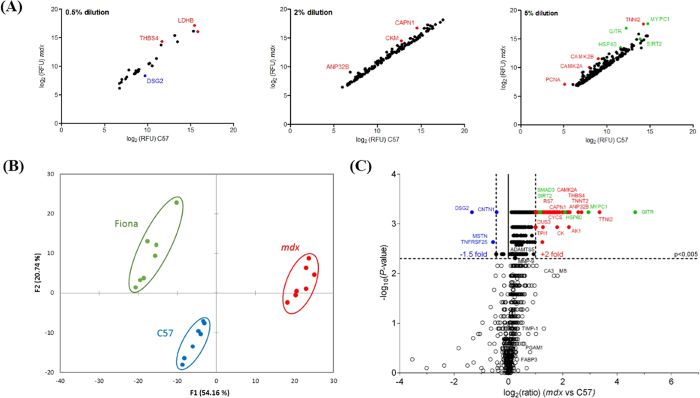
Identification of protein biomarkers in dystrophic serum. Serum samples from C57, mdx and Fiona mice were analysed using the SOMAscan methodology. (**A**) Scatter plots showing abundance of differentially expressed protein by dilution groups. (**B**) Principal components analysis (the first two components representing 74.9% of the data are shown). (**C**) Volcano plot visualizing significant protein changes in mdx serum determined by Mann-Whitney U test (p < 0.005). Red indicates up-regulated protein (>2.0 fold) previously described as potential mdx/DMD serological biomarkers. Blue indicates down-regulated proteins (<0.70 fold) and green are newly discovered markers with a >2.0 fold abundance in dystrophic serum.

We first compared C57 and *mdx* animals and identified 83 serum protein significantly changed in abundance by at least 2 fold difference (Mann-Whitney U test, FDR correction, P < 0.005, q < 0.01) (Table S1). Levels of a large majority of these markers were upregulated (79; >2 fold) and abundance of 4 serum protein were significantly decreased (CNTN1, Contactin-1; TNFRSF25, Tumor necrosis factor receptor superfamily member 25; MSTN, Myostatin; DSG2, Desmoglein-2; <0.7 fold). As previously reported in the *mdx* model^55^,^56^,^61^ and in DMD patients^55^,^56^, levels of serological proteins with statistically significant changes in abundance are increased rather than decreased. Analysis of their abundance showed that a greater number of these highly differential *mdx* markers were identified from the highest 5% dilution group and are expressed at a low abundance, whereas only a few highly abundant markers were identified (LDHB, Lactate dehydrogenase B; THBS4, Thrombospondin 4 and DSG2, Desmoglein- 2) (Fig. 1A). A clear separation between dystrophic and healthy animals was noted using principal component analysis (Fig. 1B) suggesting that serum protein could be used to differentiate these experimental groups. Both highly differentially expressed and statically significant factors were identified by Volcano plot (Fig. 1C). Among the serological level of these 83 highly significantly expressed or repressed factors, most are muscle leakage proteins which have been previously reported to be elevated in DMD boys^55^,^56^ and *mdx* mice^55^,^56^,^61^ relative to healthy volunteers and wild-type animals respectively. Notably, we confirm that at 7 weeks of age, levels of circulating protein biomarkers linked to muscle function (MB, Myoglobin; TNNI2, Troponin I, fast skeletal muscle), metabolic dysregulation (LDHB, L-lactate dehydrogenase; TPI1, Triosephosphate isomerase; CYCS, Cytochrome C), calcium metabolism (CAMK2A, CAMK2B, Calcium/calmodulin-dependent protein kinase type II subunit alpha/beta; CAPN1, Calpain) and extracellular matrix remodelling/fibrogenesis (THBS4, Thrombospondin-4) are highly and significantly increased in *mdx* animals. We also identified several potential new serum biomarkers, notably GITR/TNFRSF18 (glucocorticoid-induced TNFR-related protein; Tumor necrosis factor receptor superfamily member 18) showing a 25.4 fold enrichment in dystrophic serum. Among these serological biomarkers identified, we also demonstrate high serological level for MYBPC1 (Myosin Binding Protein C, Slow Type; 7.7x), HSP60 (Heat shock protein 60; 3.7x), SIRT2 (NAD-dependent deacetylase sirtuin-2; 2.2x), SMAD3 (Mothers against decapentaplegic homolog 3; 2.1x) and a significant decrease in abundance for CNTN1 (0.7x), MSTN (0.7x) and DSG2 (0.4x).

### Definition of top candidate protein biomarkers for utrophin based strategies

Statistical analysis including all experimental groups identified 89 proteins highly differentiated in sera of C57, *mdx* and Fiona animals (Kruskal-Wallis one-way ANOVA, P < 0.005, q < 0.01) of which 78 showed at least a 2 fold increase and 11 at least a 0.7 fold decrease in *mdx* sera abundance (Table S2). Importantly, a very uniform overlap between Mann-Whitney U test and Kruskal-Wallis one-way ANOVA was noted and revealed a total of 80 common protein markers (76 > 2.0; 4 < 0.70) (Table 1).

**Figure 2:**
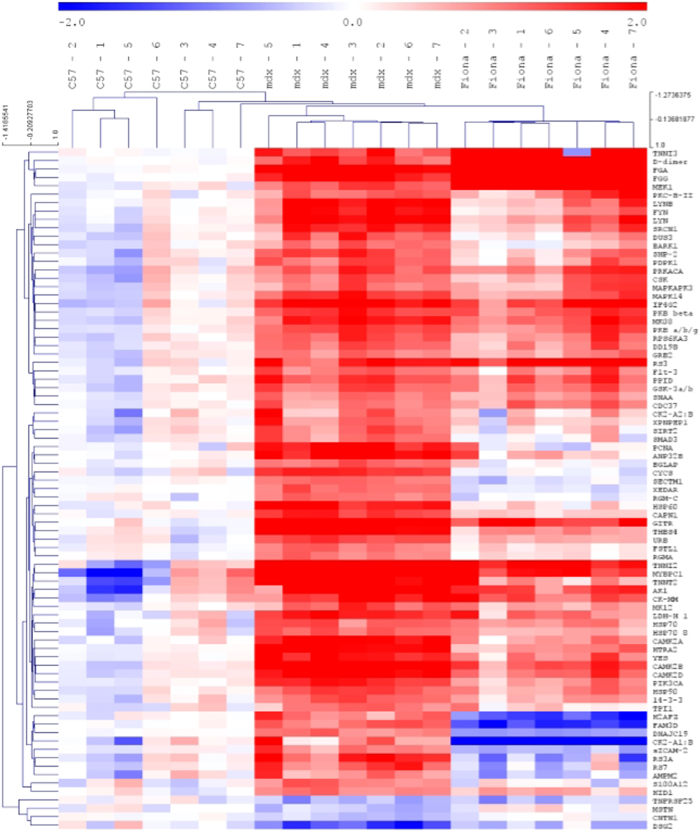
Wild-type, *mdx* and Fiona protein marker profiles. High significant protein changes determined by Mann-Witney U test and Kruskal-Wallis one-way ANOVA (*p* < 0.005, *q* < 0.01) were analysed by hierarchical clustering in all experimental groups. Red indicates up-regulated proteins and blue indicates down-regulated proteins.

**Figure 3:**
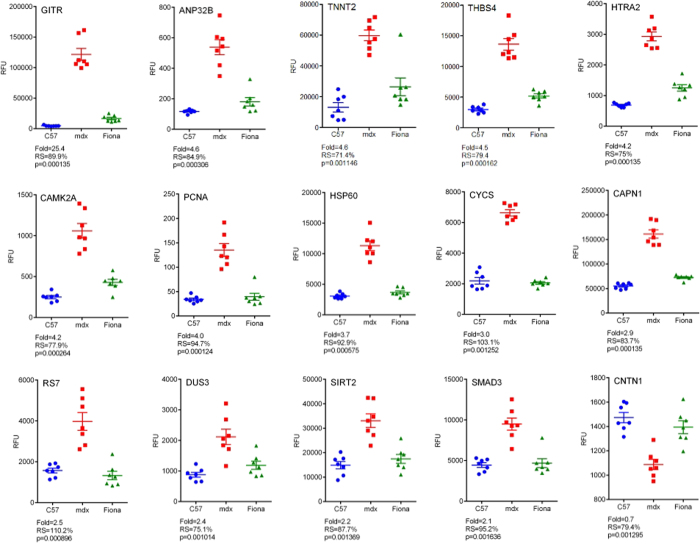
Top candidate biomarkers in dystrophic and Fiona serum. Protein abundance per individual biological replicate was plotted for the top 15 ranked candidate biomarkers identified by SOMAscan. *mdx* vs C57 fold changes, Recovery score (RS) in Fiona compared to *mdx* and C57, Kruskal-Wallis one-way ANOVA *p* values are indicated for each protein. Error bars indicate mean +/− SEM.

To appreciate potential rescue of these serological biomarkers in transgenic *mdx* Fiona animals expressing high levels of utrophin, we used the Recovery score, a common, quantitative and comparative scoring system^36^,^62^ (Table 1). This score is based on the measurements of the parameters studied on three different specimens and calculation follows the equation: RS = (“treated” − “untreated”)/(“normal” − “untreated)*100] = [(Fiona − *mdx*)/(C57 − *mdx*) * 100]. The recovery score ranges from 0%, when the increased utrophin in Fiona mice has no effect to 100% when the increased utrophin in Fiona animals display the same parameter value as the wild-type one. To rank serological biomarkers, thresholds were arbitrary defined following these criteria: (+++), RS ≥ 70, restored to wild-type levels; (++), 50 ≤ RS > 70, restored towards wild-type levels; (+), 25 ≤ RS > 50, low and inconsistent restoration towards wild-type; (−), 0 ≤ RS > 25, not restored towards wild-type levels. From the 80 serum markers previously defined, more than 75% were partially or fully rescued towards a wild-type level in Fiona mice due to high levels of utrophin. Hierarchical clustering illustrates profiles of wild-type, dystrophic and high utrophin context (Fig. 2). We next defined a set of 15 candidates (Table 2) as the most promising candidate DMD biomarkers for utrophin based strategies based on the following criteria: (i) differentially abundant with a 2 fold difference, (ii) statistically significant (p < 0.005) as determined by Kruskal-Wallis one-way ANOVA and Mann-Whitney U tests, (iii) recovery score >70% in transgenic *mdx* Fiona mice expressing high content of utrophin, (iv) previously described as potential biomarkers in DMD patients and/or *mdx* animals, (iv) known to be associated with pathways involved in modulation of utrophin expression and (v) function and protein group to obtain an homogeneous and representative panel of robust biomarkers. Table 2 and Fig. 3 present results in C57, *mdx* and Fiona mice for these 15 biomarkers candidates all rescue in high utrophin context. Importantly, Hathout *et al*., recently reported ANP32B (Acidic Nuclear Phosphoprotein 32 Family Member B), CAMK2A, RS7 (40S ribosomal protein S7), CYCS, THBS4 as serological proteins significantly increased in DMD patients^55^,^56^. TNNT2 (Troponin T, cardiac muscle) was also recently reported as increased in Becker^63^ and Duchenne patients and carriers^64^. In *mdx* mice, abundance of HTRA2 (Serine protease HTRA2, mitochondrial), PCNA (Proliferating cell nuclear antigen), DUS3 (Dual specificity protein phosphatase 3), CAPN1 as ANP32B, CAMK2A, THBS4 and CYCS is enriched in dystrophic sera^61^. Alongside these described markers, we have included five new undescribed potential circulating protein biomarkers in this panel: GITR/TNFRSF18, serum protein with the highest deregulation observed in this study (25.4x); SIRT2, a potential therapeutic avenue involving key utrophin pathways^65^ CNTN1, a neural adhesion and neuromuscular junction protein significantly reduced in dystrophic serum^66^ and two factors previously described as increased in muscle biopsies of DMD patients^67^ and *mdx* tissue^68^ but not yet in blood: HSP60 and a key intracellular signalling mediator for both transforming growth factor-β and myostatin, SMAD3. All these biomarker candidates are rescued in the high utrophin context of the Fiona mouse (Table 2, Fig. 3) and therefore represent interesting markers to evaluate benefits of utrophin based strategies.

**Figure 4:**
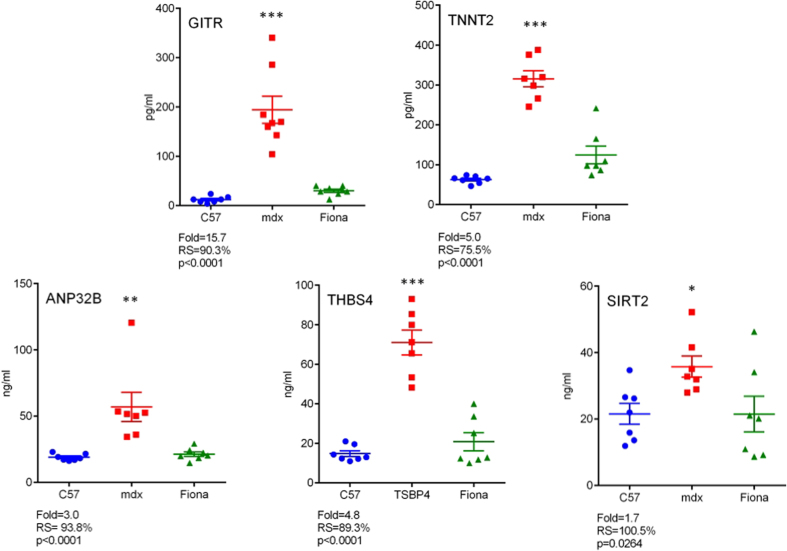
ELISA confirmation of candidate biomarkers. Five of the top candidate serological marker proteins were validated by ELISA. Individual biological replicates are shown and *mdx* vs C57 fold changes, Recovery score in Fiona compared to *mdx* and C57 and Kruskal-Wallis one-way ANOVA *p* values are indicated for each protein. Bonferroni *post hoc* test significance values are indicated as (*)p < 0.05; (**)p < 0.01 and (***)p < 0.001. Error bars indicate mean +/− SEM.

Finally, we confirmed a subset of five serological markers by ELISA to define an homogeneous set of markers based on function and pathophysiological features including muscle function (TNNT2), extracellular matrix remodelling (THBS4), immune response (GITR) and metabolic regulation (SIRT2). The multifunctional protein ANP32B was also included in this panel. Despite some small discrepancies, protein abundance was found to be significantly enriched in *mdx* serum relative to wild-type controls for all these targets (Fig. 4). In *mdx* transgenic Fiona mouse expressing a high level of utrophin, the levels of all serological markers were restored toward C57 levels as previously noted in the SOMAscan assay. CAMK2A protein was not detectable by ELISA. Details for the ELISA are specified in Table S3.

## Discussion

The effective execution of clinical trials in DMD has been severely hampered by the lack of robust biomarkers. In the present study, we have addressed the development of biomarkers for utrophin modulation. Ezutromid, a drug which shows efficacy in the *mdx* mouse has recently entered clinical trials and biomarkers are needed to facilitate these trials and provide robust end points. We utilised an aptamer-based proteomic screening approach to profile candidate biomarkers in serum of 7 weeks old C57, *mdx* and Fiona (*mdx* transgenic overexpressing high level of utrophin) mice. This technology, complementary to mass spectrometry and antibody-based arrays previously used with success to investigate serum protein abundance in DMD patients^56^ and dystrophic animals^61^, measures concentration of a higher number of predetermined 1,310 proteins with high affinity. Whereas some putative serological markers as F13A1, MYOM3 or TNNT3, are absent from the SOMAscan assay, SOMAmer reagents were defined to bind to recombinant human protein. Consequently, potential interspecies differences in protein sequences may alter results obtained with the SOMAscan platform and murine samples.

In our study, analysis of dystrophic sera revealed a clear separation between dystrophic and healthy animals, and 83 circulating protein in *mdx* sera, mostly of muscle origin, were significantly altered with a 2 fold change in abundance compared to wild-type animal. A majority of the identified factors - TNNI2, CAMK2A/B, ANP32B, THBS4, PCNA, CKM - were previously described in DMD boys and in *mdx* mouse^55^,^56^,^61^,^69^, confirming a rich set of common protein biomarkers to assess pre-clinical and clinical studies and supporting the usefulness of the SOMAscan assay as a discovery biomarker platform. Importantly, levels of some well-documented biomarkers, previously described at 12-weeks of age (PGAM1, TIMP-1, CA3 or ADAMTS5), were not significantly increased in our study with 7 weeks old dystrophic animals, suggesting an important age dependence for these markers, only deregulated at later stages of the pathology. Similar to the study performed by Hathout *et al*. with 2 months old animals^55^, we observed a significantly high induction of THBS4, CYSC or CKM in 7 weeks old *mdx* animals. Nevertheless, in our study, serological level of FABP3 was not deregulated. Additionally, we identified GITR, MYPC1, HSP60, SMAD3, SIRT2, CNTN1 and MSTN as new potential putative serum dystrophic markers. Notably, in our study, GITR, a co-stimulatory immune checkpoint molecule, and member of the tumour necrosis factor receptor (TNF-R) superfamily, induced with activation of T cells^70^, is the most deregulated factor with a 25 fold enrichment in dystrophic serum. Activated immune cell infiltrates (e.g., T lymphocytes and macrophages) are evident during early disease stages in dystrophic muscle and play a critical role in muscle wasting^71^. Thus, serum level of GITR, unchanged in previous studies with 12 weeks old *mdx* mice^55^,^61^, may be a potent marker for early dysregulation of immune system in Duchenne muscular dystrophy. Interestingly, we noted significant changes in the abundance of other members of the Tumor necrosis factor receptor superfamily as XEDAR/TNFRSF27 (2.0x), TNR4/TNFRSF4, TWEAKR/TNFRSF12A, BAFF Receptor/TNFRSF13C, RANK/TNFRSF11A, OPG/TNFRSF11B (1.4x; *data not shown*) and DR3/TNFRSF25 (0.7x). RELT/ TNFRSF19 was previously described as significantly decreased in DMD patient^56^ highlighting importance of this group of cytokine receptors primarily involved in pleatoric activities as inflammation, apoptosis, proliferation, survival and differentiation. Another intriguing protein that emerged from our study is the neural immunoglobulin family adhesion molecule Contactin-1, significantly reduced in abundance in *mdx* sera. CTNT1 was previously documented as expressed in the central and peripheral nervous system^72^ and at the neuromuscular junction in skeletal muscle^66^. Interestingly, CTNT1 deficiency was associated to the Compton-North congenital myopathy (CNCM) [MIM:612540], a familial lethal form of congenital myopathy, inherited in an autosomal-recessive fashion and characterized by ataxia, progressive muscle weakness and postnatal lethality^66^. It was proposed that loss of contactin-1 could impair communication or adhesion between nerve and muscle, resulting in severe myopathic phenotype. Interestingly, level of Contactin-5 was previously described as significantly decreased in serum from DMD patients^56^. Another protein of interest, significantly reduced in our study, is Growth Differentiation Factor 8/Myostatin (GDF8/MSTN), a member of the transforming growth factor beta (TGFβ) superfamily, acting as a major negative regulator of skeletal muscle mass^73^. MSTN and approaches to limit the activity of this secreted factor have been under extensive investigation for decades in dystrophic animal models^74^ and DMD patients^19^. Several reports failed to show induction of myostatin in DMD^75^,^76^ and in correlation with our findings, two studies reported a marker fourfold down-regulation of myostatin in *mdx* mice^77^,^78^. As myostatin abundance may not reflect myostatin activity, and that common adaptation of the myostatin level did not occur in dystrophic muscles, the usefulness of MSTN as serological biomarker can be questioned. Furthermore, GDF11 (unchanged in our study), highly homologous to myostatin, was recently reported as reduced in serum from DMD patients^56^. Whereas further studies are required to address the therapeutic potential of the balance GDF8 (inhibition)/11 (upregulation), a possible limitation of the SOMAscan assay is the cross-reactivity of SOMAmer reagents with closely homologous proteins.

To define a panel of robust serological biomarkers for utrophin modulation strategies, we next compared levels of these proteins in C57, *mdx* and Fiona sera. A first essential point to note is the specific context of the *mdx* transgenic Fiona mice expressing a high level of utrophin. These mice benefit from constant utrophin upregulation during developmental stages and after birth, and are histologically and functionally indistinguishable from wild-type animals^35^. Whereas systemic oral treatment with utrophin modulators aim to correct the pathology at later stages of the disease in animal models^21^,^37^ as DMD patients^39^, the disease is delayed from the initial stages in Fiona mice. Thus, this high level utrophin context serves as positive control for *mdx* mice treated with small utrophin inducers. Whereas further analysis will be required to define the sensitivity and robustness of potential serological biomarkers in therapies aiming to overexpress utrophin in pre-clinical and clinical settings, it is expected that the recovery of serological marker levels is dependent on the levels of induced utrophin expression after drug treatment. Among the initial 1,310 serum proteins analysed, a statistical analysis including all experimental groups, revealed 89 proteins highly differentiated in sera of C57, *mdx* and Fiona mice. Interestingly, a very positive correlation was noted between these markers and the previous panel of 83 serological protein biomarkers defined in *mdx* mice compared to wild-type. From the subsequent set of 80 common protein markers, profiles of these three distinct genotype/phenotypes showed a clear separation supporting the usefulness of monitoring the effectiveness of therapeutic interventions with utrophin modulators. Using a recovery score, we observed that more than 75% of the defined serological proteins were restored towards wild-type in Fiona mice, due to high levels of utrophin. However, the serum biomarker profile of the Fiona mice is not identical to the wild-type animal, suggesting that the biomarker rescue is either not complete or not necessary to obtain significant histological and functional benefits. Among these 80 markers, some were previously described in *mdx* mice as fully or partially rescued after dystrophin restoration^61^. These serum biomarkers are of interest as they may provide a common and comparative panel of serum biomarkers to therapeutic strategies aiming to rescue the sarcolemma stability by rescuing dystrophin or increasing utrophin expression.

More interesting are the unique markers specific to each strategy. A previous study showed that levels of TNNI3 (Troponin I, cardiac muscle) but not DUS3 and TPI1 were rescued in response to dystrophin restoration^61^. In Fiona mice, levels of DUS3 and TPI1 are normalised toward wild-type level, whereas TNNI3 is unchanged. Despite different contexts and the need for further studies, these results could emphasise specific serum biomarkers for dystrophin and utrophin strategies. As the first difference between utrophin and dystrophin is the specific spatio-temporal expression of each protein^26^, it is very likely utrophin fulfils specific roles and actions different from dystrophin, and *vice versa,* and that benefits of high level of utrophin may not only be restricted to improvement due to stabilisation of the muscle membrane. Thus, all these proteins may provide important insights in roles of utrophin protein and undiscovered benefits of high utrophin levels.

Importantly in our definition of robust biomarkers for utrophin modulation strategies, 34 serological protein markers were fully rescued to C57 level in Fiona mice. A set of 15 candidates was therefore defined. We noted the full restoration to wild-type level of previously biomarkers described as enriched in DMD (ANP32B, TNNT2, THBS4, CAMK2A, RS7, CYCS,) and *mdx* (HTRA2, PCNA, SMAD3, CAPN1, DUS3). Importantly, some of the common DMD associated biomarkers such as CK and TNNI3 are not included in the top 15 utrophin responsive biomarker candidates as they are poorly recovered in *mdx* transgenic Fiona mice (Table S2). Furthermore, despite a high increased level in *mdx* animals and a full rescue in Fiona mice, myoglobin was not selected as this marker does meet initial statistical significance criteria (Table S2). We also identified new *mdx* biomarkers as GITR, HSP60, SMAD3, SIRT2 and Contactin-1, all fully rescued in Fiona mice. Finally, despite some minor discrepancies, five top candidate protein biomarkers (ANP32B, THBS4, TNNT2, GITR and SIRT2) were validated by ELISA. Of great interest is the NAD-dependent deacetylase sirtuin-2 (SIRT2), a cytoplasmic enzyme involved in a large range of phenomenon as metabolic homeostasis, microtubule reorganisation, inflammatory responses and mitochondrial biogenesis^65^, notably by deacetylating PGC-1α, well known to induce utrophin expression^79^. SIRT2 is an emerging target in neurodegenerative diseases as inhibition of this sirtuin was beneficial. Interestingly, overexpression of SIRT1, another member of the sirtuin family, was recently reported to increase utrophin expression and ameliorate pathophysiology in *mdx* mouse^80^,^81^. Pharmacological modulation of the SIRT1/SIRT2 balance is therefore an interesting avenue for DMD.

In conclusion, we have identified several serological protein biomarkers to assist in the development of utrophin modulation strategies for DMD. Among 1,310 proteins, we progressively selected well-documented and undiscovered *mdx* markers, all rescued in the *mdx* transgenic Fiona mice. We therefore defined a final panel of 15 therapeutic monitoring biomarkers and confirmed five markers, easily measured in an automated fashion by ELISA. This work may help in the evaluation of Ezutromid currently in a clinical phase 2 trials and should accelerate development of future generations of utrophin modulators for a more rapid translation to DMD patients.

## Methods

### Animal samples

All animal procedures were performed in accordance with UK Home Office regulations which conform with the European Community Directive published in 1986 (86/609/ EEC). The work was performed under certificate of designation number 30/2306 and project license number 30/3104 following approval by the University of Oxford Departments of Physiology, Anatomy & Genetics and Experimental Psychology Joint Departmental Ethics Review Committee. 7 week old male C57/Bl10, C57/Bl10ScSn-*Dmdmdx*/J (*mdx*) and C57/Bl10ScSn-*Dmdmdx*/J-Tg(ACTA1-Utrn)2Ked (Fiona, Fio) mice (*n* = 7) were sacrificed. Each mouse used in this study was pentobarbital and blood immediately collected from the jugular vein and process according to the recommended Sample Handling and Processing SSM-001 Rev 5 Sop (SomaLogic, Inc). Briefly, whole blood was allowed to clot for 60 minutes at room temperature prior to centrifugation 2200 × g for 15 minutes. Typically 400 ul of blood could be collected per mouse aged from 7 weeks and 140 ul of serum obtained. Each sample was then aliquot in 70 ul volume and store at −80 °C for proteome profiling as described below. After blood collection, muscle samples were frozen in liquid nitrogen-chilled isopentane, and stored at −80 °C.

### SOMAscan Assay

Serum proteome profiling was performed at SomaLogic, Inc (Boulder, CO, USA) using the 1,310-plex SomaScan platform. In this assay, each protein is targeted by a unique SOMAmer (Slow Off-rate Modified Aptamer) - a chemically modified nucleic acid ligand - attached to streptavidin beads via a tail consisting of a Cyanine3 fluorophore used for detection and quantification, a photocleavable linker and a photo-cleavable biotin molecule in order to capture specific proteins of interest. For each samples, three dilutions (0.5%, 2% and 5%) were prepared with a set of SOMAmers complexes and immobilised on streptavidin-coated beads. Once bound and after washes to reduce non-specific binding, proteins remain captured are biotinylated using NHS-PE04-Biotin and specifically photocleaved by ultraviolet light to releases the SOMAmer-biotinylated protein complexes. Biotin-labelled proteins-SOMAmers are then bound to a different set of streptavidin beads, washed to remove free SOMAmers and precipitated. Using a high-pH denaturing wash, SOMAmers are removed from their protein targets and eluted to be finally quantified using standard DND microarrays. Samples were randomly assigned to plates. Quality control procedures with intra-run normalisation and inter-run calibration were performed according to the SomaLogic good laboratory practice quality system (SSM-020 Rev 3). All samples passed quality control criteria for biases in SOMAmer hybridization.

### ELISA

Enzyme-linked immunosorbent assay (ELISA) kits were purchased from antibodies-online (Aachen, Germany) and run to validate selected candidate serum protein biomarkers. ELISA kits used were: Acidic (Leucine-Rich) Nuclear phosphoprotein 32 Family, Member B, ANP32B (ABIN1745100); Calcium/calmodulin-Dependent Protein Kinase II alpha, CAMK2A, (ABIN426487); Cardiac Troponin T2 ELISA Kit, TNNT2, (ABIN426397); Tumor Necrosis Factor Receptor Superfamily, Member 18, TNFRSF18/GITR, (ABIN1672796); Sirtuin 2, SIRT2 (ABIN1144096), and Thrombospondin 4, THBS4 (ABIN426909). Sera of animals involved in the SOMAscan assay were used and diluted to fall within the linear range of each respective assay. Due to limited amount of sample, additional age matched animals were included in the study. ELISAs were performed according to manufacturer’s instructions. Samples concentrations were calculated using a four parameter logistic (4-PL) curve fit of the standard curves with MasterPlex 2.0.0.76 software.

### Statistics

SOMAscan proteomic data reported in relative fluorescence units (RFU) were analysed using the SOMAsuite analysis software (V1.0.3). To identify differentially expressed protein between C57 and *mdx* animals, Mann-Witney U test (two-sided) tests was used. Comparison of C57, *mdx* and Fiona groups were performed using Kruskal-Wallis one-way ANOVA in correlation with Mann-Witney U test. Non-parametric tests were used as Shapiro-Wild test (GraphPad Prism 6.01) defined data as not normally distributed. Heatmap visualisation and hierarchical clustering were performed using log-transformed RFU and MeV (Multiple Experiment Viewer 4.9.0; The Institute for Genomic Research, Rockville, MD, USA)^82^. Principal Component Analysis (PCA) was studied using XLSTAT 2014.5.03. Using SOMAscan values, the percentage recovery score was calculated as described on the TREAT-NMD M. 1.1_001 SOP. Additional statistical analyses were performed using GraphPad Prism 6.01 software (GraphPad Software Inc, La Jolla, CA) as one-way ANOVA, Bonferroni post hoc test. Data are presented as mean ± SEM (standard error of mean), with n indicating the number of independent biological replicates used in each group for comparison. Differences were considered significant at (*) p < 0.05; (**)p < 0.01 and (***)p < 0.001.

## Additional Information

**How to cite this article**: Guiraud, S. *et al*. Identification of serum protein biomarkers for utrophin based DMD therapy. *Sci. Rep.* **7**, 43697; doi: 10.1038/srep43697 (2017).

**Publisher's note:** Springer Nature remains neutral with regard to jurisdictional claims in published maps and institutional affiliations.

## Supplementary Material

Supplementary Data

## Figures and Tables

**Table 1:** Serum *mdx* protein biomarkers and rescue in *mdx* transgenic Fiona mice.

Rank	Protein	Target	Uniprot	Dilution	Fold change (mdx vs C57)	pValue	Recovery score (Fiona)
1	Tumor necrosis factor receptor superfamily member 18	TNFRSF18/GITR	Q9Y5U5	5	25.4	0.0001	+++
2	Troponin I, fast skeletal muscle	TNNI2	P48788	5	10.2	0.0002	++
3	Myosin-binding protein C, slow-type	MYBPC1	Q00872	5	7.7	0.0003	++
4	Fibrinogen	FGA	P02671	0.5	6.5	0.0001	−
5	Calcium/calmodulin-dependent protein kinase type II subunit beta	CAMK2B	Q13554	5	5.9	0.0001	++
6	Fibrinogen gamma chain	FGG	P02679	0.5	4.8	0.0001	−
7	Adenylate kinase isoenzyme 1	AK1	P00568	2	4.7	0.0007	++
8	Acidic leucine-rich nuclear phosphoprotein 32 family member B	ANP32B	Q92688	2	4.6	0.0003	+++
9	Troponin T, cardiac muscle	TNNT2	P45379	5	4.6	0.0011	+++
10	Thrombospondin-4	THBS4	P35443	0.5	4.5	0.0002	+++
11	Calcium/calmodulin-dependent protein kinase type II subunit delta	CAMK2D	Q13557	5	4.5	0.0001	++
12	Serine protease HTRA2, mitochondrial	HTRA2	O43464	5	4.2	0.0001	+++
13	Calcium/calmodulin-dependent protein kinase type II subunit alpha	CAMK2A	Q9UQM7	5	4.2	0.0003	+++
14	Tyrosine-protein kinase Lyn	LYN	P07948	5	4	0.0003	++
15	Tyrosine-protein kinase Lyn, isoform B	LYN	P07948	5	4	0.0003	+++
16	Proliferating cell nuclear antigen	PCNA	P12004	5	4	0.0012	+++
17	Eukaryotic translation initiation factor 4 gamma 2	EIF4G2	P78344	5	4	0.0012	−
18	60 kDa heat shock protein, mitochondrial	HSP60	P10809	5	3.7	0.0006	+++
19	Tyrosine-protein kinase Fyn	FYN	P06241	5	3.5	0.0004	+++
20	Tyrosine-protein kinase Yes	YES1	P07947	5	3.5	0.0003	++
21	Creatine kinase M-type	CKM	P06732	2	3.5	0.0011	+
22	Mitogen-activated protein kinase 8	MAPK8	P45983	5	3.4	0.0007	+
23	L-lactate dehydrogenase B chain	LDHB	P07195	0.5	3.3	0.0005	++
24	40S ribosomal protein S3	RPS3	P23396	5	3.2	0.0005	−
25	PIK3CA/PIK3R1	PIK3CA PIK3R1	P42336	5	3.1	0.0001	++
26	40S ribosomal protein S3a	RPS3A	P61247	5	3.1	0.0006	+++
27	Cytochrome c	CYCS	P99999	5	3	0.0013	+++
28	Proto-oncogene tyrosine-protein kinase Src	SRC	P12931	5	3	0.0007	++
29	D-dimer	FGA FGB FGG	P02671	0.5	2.9	0.0001	−
30	Calpain I	CAPN1	P07384	2	2.9	0.0001	+++
31	RAC-beta serine/threonine-protein kinase	AKT2	P31751	5	2.8	0.001	−
32	Mitogen-activated protein kinase 14	MAPK14	Q16539	2	2.8	0.0005	+
33	Peptidyl-prolyl cis-trans isomerase D	PPID	Q08752	5	2.8	0.0008	−
34	Troponin I, cardiac muscle	TNNI3	P19429	5	2.8	0.0036	−
35	RAC-alpha/beta/gamma serine/threonine-protein kinase	AKT1 AKT2 AKT3	P31749	2	2.7	0.0007	−
36	Coiled-coil domain-containing protein 80	CCDC80	Q76M96	2	2.7	0.0002	++
37	Tyrosine-protein phosphatase non-receptor type 11	PTPN11	Q06124	5	2.6	0.001	++
38	40S ribosomal protein S7	RPS7	P62081	5	2.5	0.0009	+++
39	Heat shock protein HSP 90-alpha/beta	HSP90AA1/AB1	P07900	2	2.5	0.0004	+
40	Ribosomal protein S6 kinase alpha-3	RPS6KA3	P51812	5	2.5	0.0005	+
41	cAMP-dependent protein kinase catalytic subunit alpha	PRKACA	P17612	5	2.5	0.0012	−
42	Dual specificity mitogen-activated protein kinase kinase 1	MAP2K1	Q02750	5	2.4	0.0001	−
43	Dual specificity protein phosphatase 3	DUSP3	P51452	5	2.4	0.002	+++
44	Heat shock 70 kDa protein 1A	HSPA1A	P0DMV8	5	2.4	0.0004	+
45	Casein kinase II 2-alpha’:2-beta heterotetramer	CSNK2A2 CSNK2B	P19784	5	2.4	0.0043	+++
46	Glycogen synthase kinase-3 alpha/beta	GSK3A GSK3B	P49840	5	2.4	0.0011	−
47	Mitogen-activated protein kinase 12	MAPK12	P53778	5	2.4	0.0004	++
48	3-phosphoinositide-dependent protein kinase 1	PDPK1	O15530	5	2.3	0.002	++
49	Heat shock cognate 71 kDa protein	HSPA8	P11142	2	2.3	0.0003	++
50	ATP-dependent RNA helicase DDX19B	DDX19B	Q9UMR2	5	2.3	0.0006	+
51	14–3–3 protein family	YWHAB	P31946	2	2.3	0.0003	++
52	Protein FAM3D	FAM3D	Q96BQ1	5	2.2	0.0001	+++
53	NAD-dependent protein deacetylase sirtuin-2	SIRT2	Q8IXJ6	5	2.2	0.0014	+++
54	Xaa-Pro aminopeptidase 1	XPNPEP1	Q9NQW7	5	2.2	0.0008	+++
55	Receptor-type tyrosine-protein kinase FLT3	FLT3	P36888	5	2.2	0.0018	+
56	Protein kinase C beta type (splice variant beta-II)	PRKCB	P05771	2	2.2	0.0016	−
57	Tumor necrosis factor receptor superfamily member 27	EDA2R	Q9HAV5	5	2.2	0.0001	+++
58	Osteocalcin	BGLAP	P02818	2	2.2	0.0012	+++
59	Histone H2A.z	H2AFZ	P0C0S5	5	2.2	0.0001	+++
60	Tyrosine-protein kinase CSK	CSK	P41240	2	2.1	0.0027	−
61	Mothers against decapentaplegic homolog 3	SMAD3	P84022	5	2.1	0.0016	+++
62	Secreted and transmembrane protein 1	SECTM1	Q8WVN6	2	2.1	0.0003	+++
63	Follistatin-related protein 1	FSTL1	Q12841	2	2.1	0.0004	+++
64	beta-adrenergic receptor kinase 1	ADRBK1	P25098	5	2.1	0.0012	+++
65	Hemojuvelin	HFE2	Q6ZVN8	2	2	0.001	+++
66	Intercellular adhesion molecule 2	ICAM2	P13598	2	2	0.0001	+++
67	Alpha-soluble NSF attachment protein	NAPA	P54920	2	2	0.0006	+
68	Protein S100-A12	S100A12	P80511	2	2	0.0013	+++
69	MAP kinase-activated protein kinase 3	MAPKAPK3	Q16644	2	2	0.0031	−
70	Repulsive guidance molecule A	RGMA	Q96B86	2	2	0.0005	+++
71	Mitochondrial import inner membrane translocase subunit TIM14	DNAJC19	Q96DA6	5	2	0.0001	+++
72	Triosephosphate isomerase	TPI1	P60174	2	2	0.0034	++
73	Growth factor receptor-bound protein 2	GRB2	P62993	5	2	0.0008	−
74	Hsp90 co-chaperone Cdc37	CDC37	Q16543	5	2	0.001243	−
75	Methionine aminopeptidase 2	METAP2	P50579	5	2	0.00098	+++
76	Nidogen-1	NID1	P14543	2	2	0.000276	+
77	Contactin-1	CNTN1	Q12860	0.5	0.7	0.001295	+++
78	Growth/differentiation factor 8	MSTN	O14793	0.5	0.7	0.003311	+++
79	Tumor necrosis factor receptor superfamily member 25	TNFRSF25	Q93038	5	0.7	0.00498	−
80	Desmoglein-2	DSG2	Q14126	0.5	0.4	0.000456	++

High significant increased and decreased protein were defined using Mann-Witney U test and Kruskal-Wallis one-way ANOVA (p < 0.005; q < 0.01). (+++) restored to wild-type levels (recovery score ≥70%), (++) restored towards wild-type levels (50≤ recovery score <70, (+) low and inconsistent restoration towards wild-type (25≤ recovery score <50), (−) not restored towards wild-type levels (0≤ recovery score <25).

**Table 2:** Selection of serum protein biomarkers for utrophin based DMD therapy.

Rank	Protein	Target	Uniprot	Groups	Dilution	Fold change (mdx vs C57)	pValue	Recovery score (Fiona)	Reference
1	Tumor necrosis factor receptor superfamily member 18	**GITR**	Q9Y5U5	Immune response	5	25.4	0.000135	89.9	
8	Acidic leucine-rich nuclear phosphoprotein 32 family member B	**ANP32B**	Q92688	Multifunctional/Other	2	4.6	0.000306	84.9	55, 56, 61
9	Troponin T, cardiac muscle	**TNNT2**	P45379	Muscle function	5	4.6	0.001146	71.4	63, 64
10	Thrombospondin-4	**THBS4**	P35443	ECM remodeling	0.5	4.5	0.000162	79.4	61
12	Serine protease HTRA2, mitochondrial	HTRA2	O43464	Metabolism (Mitochondria)	5	4.2	0.000135	75	61
13	Calcium/calmodulin-dependent protein kinase type II subunit alpha	**CAMK2A**	Q9UQM7	Metabolism (Calcium)	5	4.2	0.000264	77.9	61
15	Tyrosine-protein kinase Lyn, isoform B	LYN	P07948	Multifunctional/Other	5	4	0.000264	71.2	61
16	Proliferating cell nuclear antigen	PCNA	P12004	DNA repair/maintenance	5	4	0.001243	94.7	61
18	60 kDa heat shock protein, mitochondrial	HSP60	P10809	Metabolism (Mitochondria)	5	3.7	0.000575	92.9	67
19	Tyrosine-protein kinase Fyn	FYN	P06241	Immune response	5	3.5	0.000389	75.5	
26	40S ribosomal protein S3a	RS3A	P61247	Ribosomal biogenesis	5	3.1	0.000637	113.4	56
27	Cytochrome c	CYCS	P99999	Metabolism (Mitochondria)	5	3	0.001252	103.1	61
30	Calpain I	CAPN1	P07384	Calcium metabolism	2	2.9	0.000135	83.7	61
38	40S ribosomal protein S7	RS7	P62081	Ribosomal biogenesis	5	2.5	0.000897	110.2	56
43	Dual specificity protein phosphatase 3	DUS3	P51452	Multifunctional/Other	5	2.4	0.001984	75.1	61
45	Casein kinase II 2-alpha’:2-beta heterotetramer	CSNK2A2 CSNK2B	P19784	Multifunctional/Other	5	2.4	0.004261	80.8	
52	Protein FAM3D	FAM3D	Q96BQ1	Multifunctional/Other	5	2.2	0.000135	153.1	
53	NAD-dependent protein deacetylase sirtuin-2	**SIRT2**	Q8IXJ6	Metabolism (Mitochondria)	5	2.2	0.001369	85.7	
54	Xaa-Pro aminopeptidase 1	XPNPEP1	Q9NQW7	Multifunctional/Other	5	2.2	0.000779	76.8	
57	Tumor necrosis factor receptor superfamily member 27	XEDAR	Q9HAV5	Multifunctional/Other	5	2.2	0.000135	112	61
58	Osteocalcin	BGLAP	P02818	Metabolism (Calcium)	2	2.2	0.001243	96.3	
59	Histone H2A.z	H2AFZ	P0C0S5	Nucleosome structure	5	2.2	0.000135	153.3	
61	Mothers against decapentaplegic homolog 3	SMAD3	P84022	Multifunctional/Other	5	2.1	0.001636	95.2	68
62	Secreted and transmembrane protein 1	SECTM1	Q8WVN6	Immune response	2	2.1	0.000306	114.5	
63	Follistatin-related protein 1	FSTL1	Q12841	Inflammation	2	2.1	0.000402	82.2	
64	Beta-adrenergic receptor kinase 1	ADRBK1	P25098	Multifunctional/Other	5	2.1	0.001198	79.4	
65	Hemojuvelin	RGM-C	Q6ZVN8	Multifunctional/Other	2	2	0.000959	108.3	
66	Intercellular adhesion molecule 2	ICAM2	P13598	Inflammation/Immune response	2	2	0.000135	131.8	
68	Protein S100-A12	S100A12	P80511	Inflammation/Immune response	2	2	0.001252	98.5	
70	Repulsive guidance molecule A	RGMA	Q96B86	Multifunctional/Other	2	2	0.000514	83.5	
71	Mitochondrial import inner membrane translocase subunit TIM14	DNAJC19	Q96DA6	Metabolism (Mitochondria)	5	2	0.000135	141.6	
75	Methionine aminopeptidase 2	METAP2	P50579	Mutilfunctional/Other	5	2	0.00098	128.5	
77	Contactin-1	CNTN1	Q12860	Mutilfunctional/Other	0.5	0.7	0.001295	79.4	
78	Growth/differentiation factor 8	MSTN	O14793	Muscle function	0.5	0.7	0.003311	118.6	

Potential serological marker to estimate efficacy of utrophin based strategies were defined using Mann-Witney U test and Kruskal-Wallis one-way ANOVA (p < 0.005; q < 0.01). 32 biomarkers significantly increased (>2.0) in *mdx* mice presenting a high recovery score >70% were selected as Contactin-1 and Myostatin (<0.7). Targets in red were selected in the final 15 set of selected markers and targets in red bold were studied by ELISA.
