# Supplementary material for: Identification of serum protein biomarkers for utrophin based DMD therapy
Source: Sci Rep. 2017 Mar 2;7:43697. doi: 10.1038/srep43697 (PMC5333102; doi:10.1038/srep43697)
Supplement: Supplementary Data [file srep43697-s1.pdf]

Identification of serum protein biomarkers for utrophin based DMD therapy

Simon Guiraud[1]*, Benjamin Edwards[1], Sarah E. Squire[1], Arran Babbs[1], Nandini Shah[1], Adam Berg[1], Huijia Chen[1], Kay E. Davies[1]*

[1] Medical Research Council Functional Genomics Unit at the University of Oxford, Department of Physiology, Anatomy and Genetics, Oxford OX1 3PT,United Kingdom

*Co-correspondence

Address: Medical Research Council Functional Genomics Unit, Department of Physiology, Anatomy and Genetics, University of Oxford, Oxford OX1 3PT, United Kingdom

Telephone: +44 (0)1865 285880

Emails: simon.guiraud@dpag.ox.ac.uk and kay.davies@dpag.ox.ac.uk

Supplemental data - Legends

Figure S1: Utrophin increased rescue *mdx* phenotype. 7 week old C57, *mdx* and Fiona (n=7) were sacrificed and serum and muscles collected (A) H&E-stained transverse muscle sections of TA muscle in *mdx* mice showing necrotic areas and regenerating fibres compared to wild-type tissue. In Fiona mice, these pathological features are fully rescued due to high level of utrophin protein expression at the sarcolemma. Scale bar: 100 µm. (B) Western blots for utrophin protein showing a 2 fold increase in mdx related to C57 and a 4 fold increase in Fiona mice compared to *mdx*. (C) Quantification of centronucleation necrotic muscle area in EDL showing increased in *mdx* and full rescue in Fiona mice compared to the wild-type animals. (D) Necrosis quantification, increased in *mdx* compared to C57 and rescue to normal level in Fiona animals. (E). The difference in force produced between the first and fifth stretch is represented as a sensitive indicator of the resistance of the muscle to stretch-induced damage. EDL muscles were stretched at 15% of their fibre length whilst contracting tetanically. Values are mean ± SEM of n=7 per group; (*) p<0.05, (**) p<0.01, (***) p<0.001.

Figure S2: Quality control of SOMAscan data. (A) Table of all hybridization normalization and median normalization. (B) Hybridization normalization scale per experimental groups. (C) Median normalization scale factors per dilutions and groups

Table S1: Serum *mdx* protein biomarkers identified by SOMAscan. High significant increased and decreased protein were defined using Mann-Witney U test (p<0.005; q<0.01).

Table S2: Serum *mdx* protein biomarkers and rescue in *mdx* transgenic Fiona mice. High significant increased and decreased protein were defined using Kruskal-Wallis one-way ANOVA (p<0.005; q<0.01). **(**+++) restored to wild-type levels (recovery score >70%), (++) restored towards

wild-type levels (50< recovery score <70, (+) low and inconsistent restoration towards wild-type (recover score <50, (-) not restored towards wild-type levels.

Table S3: Details of the ELISA assays used in the study. All the ELISA kits were purchased from Antibodies-online.

Supplemental data – Methods

Histological analyses

Muscle samples were frozen in liquid nitrogen-chilled isopentane, and stored at -80°C. Transverse tibialis anterior (TA), were stained with Haematoxylin and Eosin solution (H&E). The Axioplan 2 Microscope System (Carl Zeiss, Germany) was used to obtain the images. The proportion of centrally nucleated fibres was determined by analysing the H&E images. Areas of necrosis were quantified based on the DMD_M.1.2.007 MDC1A_M.1.2.004 TREAT-NMD SOPS and performed with the Fiji ImageJ 1.49i software [1] on the whole TA sections.

Immunofluorescence

Frozen transverse TA muscle sections were blocked in 10% foetal bovine serum/PBS for 20 minutes, incubated with primary antibodies overnight at 4°C, washed in PBS and incubated with suitable Alexa Fluor secondary antibodies for 1 hour at room temperature. Sections were examined under an Axioplan 2 Microscope System (Carl Zeiss, Germany). The following antibodies and dilutions were used: goat monoclonal anti-utrophin (1:2000, UtroA), mouse monoclonal anti-Laminin-α2 (1:500, sc-59854, Santa Cruz Biotechnology, Inc.).

Protein analyses

Western blots were performed according to standard procedures. Briefly, muscles were homogenized in RIPA buffer (R0278, Sigma-Aldrich) supplemented with protease inhibitors (1:100; P8340, Sigma-Aldrich). 30 µg of total protein were heat-denatured for 5 minutes at 100°C before loading onto NuPAGE 3– 8% TRIS Acetate Midi Gel (Novex, Life Technologies) and transferred to PVDF membranes (Millipore). Membranes were blocked for 1 hour with 10% skimmed milk in 0.1% PBS-Tween20 and then incubated with primary antibodies in 0.1% PBS-T for 1 hour at room temperature. Primary antibodies used were: mouse monoclonal anti-utrophin (1:50, MANCHO3(84A), gift from

G.E. Morris) and goat polyclonal α-actinin Antibody (N-19) (1:30000, sc-7453, Santa Cruz). HRP-labelled secondary antibodies (Amersham) were incubated for 1 hour at room temperature. Immunoreactive bands were detected using ECL Western Blot Detection Reagents (SuperSignal West Pico Chemiluminescent Substrate, Thermo Scientific). The relative expression of the target proteins was estimated by densitometry using Actinin N-19 as references on a Xograph Compact X4 developer and quantified with the Fiji ImageJ 1.49i software.

Isolated muscle function analysis

Peak force, specific force, and force drop were measured from the extensor digitorum longus muscle of the treated and control mice. During dissection and experiments, muscles were bathed in oxygenated (95% O_2–5% CO_2) Krebs–Hensley solution composed of (mmol/l): NaCl, 118; $NaHCO_3$, 24.8, KCl, 4.75; KH_2PO_4, 1.18; $MgSO_4$, 1.18; $CaCl_2$, 2.54; glucose, 10 [2]. Contractile properties were measured as previously described [3]. In brief, isolated EDL were attached to a lever arm connected to a force transducer (model 300B); and stimulator (model 701B); the equipment was controlled using the signal interface (model 604A) and results were recorded by the DMC software (version 4.1.4; Aurora Scientific, Aurora, Ontario, Canada). The muscle was stimulated by single pulses of 0.2 milliseconds at 30 V while the optimum length (L_o) was determined. Optimum fibre length (L_f) was calculated by multiplying L_o by the predetermined fibre length to muscle length ratio of 0.44 [4]. A force–frequency curve was generated and the maximum isometric force calculated. Absolute force (P_o) are normalized to specific force (sP_o; kN/m^2) using the equation (muscle mass/L_f x1.06 (the density of mammalian muscle). Percentage force drop were calculated by comparing maximum force between the first (ECC0) and fifth eccentric (ECC5) contractions, expressed as a percentage of ECC0. The muscle was stimulated into tetanus at the frequency required to generate the P_o, while in tetanic state the muscle was stretched at a rate of one fibre length per second for 0.15 seconds, equating to a total stretch of 15% of fibre length. All data were digitized and analysed using the DMA software (version 3.2; Aurora Scientific).

Supplemental data –Figures/Tables

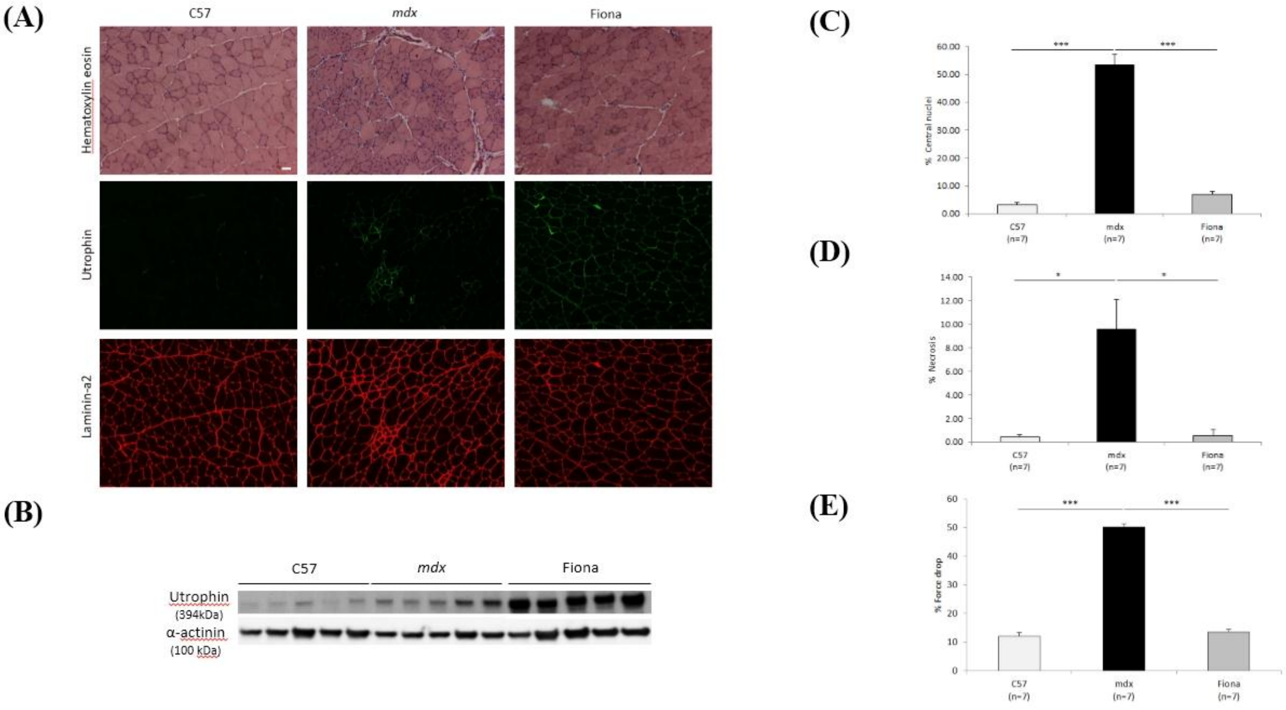

Figure S1: Utrophin increased rescue *mdx* phenotype.

(A)

Group	Sample	HybControlNormScale	RowCheck	Median Normalisation Scale Factor		
				Dilution 5%	Dilution 2%	Dilution 0.5%
C57	C57 - 1	0.958	PASS	1.058	1.035	1.009
	C57 - 2	0.993	PASS	1.037	1.06	1.033
	C57 - 3	0.903	PASS	0.954	0.929	0.957
	C57 - 4	1.011	PASS	1.009	1.018	1.018
	C57 - 5	1.12	PASS	1.044	1.064	1.026
	C57 - 6	1.001	PASS	0.938	0.931	0.977
	C57 - 7	0.956	PASS	0.935	0.936	0.967
	AVG	0.992	PASS	0.996	0.996	0.998
	STDev	0.067		0.053	0.062	0.031
mdx	mdx - 1	1.022	PASS	1.271	1.223	1.227
	mdx - 2	0.99	PASS	1.006	1.006	0.979
	mdx - 3	1.032	PASS	0.9	0.956	1.014
	mdx - 4	1	PASS	1.124	1.125	1.087
	mdx - 5	1.008	PASS	0.974	0.953	0.947
	mdx - 6	0.959	PASS	0.913	0.938	0.932
	mdx - 7	1.088	PASS	0.896	0.933	0.818
	AVG	1.014	PASS	1.012	1.019	1.001
	STDev	0.040		0.140	0.112	0.129
Fiona	Fiona - 1	1.008	PASS	0.999	0.959	1.002
	Fiona - 2	0.984	PASS	0.901	0.944	0.994
	Fiona - 3	1.022	PASS	1.175	1.207	1.034
	Fiona - 4	0.988	PASS	0.738	0.769	0.859
	Fiona - 5	0.957	PASS	1.08	1.085	1.073
	Fiona - 6	1.04	PASS	1.107	1.119	1.114
	Fiona - 7	1.118	PASS	1.012	1.033	0.931
	AVG	1.017	PASS	1.002	1.017	1.001
	STDev	0.052		0.146	0.142	0.086

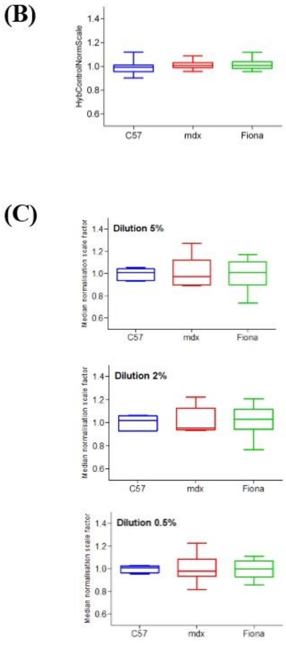

Figure S2: Quality control of SOMAscan data.

Rank	Protein	Target	Uniprot	Dilution	Fold change (mdx vs C57)	pValue
1	Tumor necrosis factor receptor superfamily member 18	TNFRSF18/GITR	Q9Y5U5	5	25.4	0.0001
2	Troponin I, fast skeletal muscle	TNNI2	P48788	5	10.2	0.0002
3	Myosin-binding protein C, slow-type	MYBPC1	Q00872	5	7.7	0.0003
4	Fibrinogen	FGA	P02671	0.5	6.5	0.0001
5	Calcium/calmodulin-dependent protein kinase type II subunit beta	CAMK2B	Q13554	5	5.9	0.0001
6	Fibrinogen gamma chain	FGG	P02679	0.5	4.8	0.0001
7	Adenylate kinase isoenzyme 1	AK1	P00568	2	4.7	0.0007
8	Acidic leucine-rich nuclear phosphoprotein 32 family member B	ANP32B	Q92688	2	4.6	0.0003
9	Troponin T, cardiac muscle	TNNT2	P45379	5	4.6	0.0011
10	Thrombospondin-4	THBS4	P35443	0.5	4.5	0.0002
11	Calcium/calmodulin-dependent protein kinase type II subunit delta	CAMK2D	Q13557	5	4.5	0.0001
12	Serine protease HTRA2, mitochondrial	HTRA2	O43464	5	4.2	0.0001
13	Calcium/calmodulin-dependent protein kinase type II subunit alpha	CAMK2A	Q9UQM7	5	4.2	0.0003
14	Tyrosine-protein kinase Lyn	LYN	P07948	5	4.0	0.0003
15	Tyrosine-protein kinase Lyn, isoform B	LYNB	P07948	5	4.0	0.0003
16	Proliferating cell nuclear antigen	PCNA	P12004	5	4.0	0.0012
17	Eukaryotic translation initiation factor 4 gamma 2	EIF4G2	P78344	5	4.0	0.0012
18	60 kDa heat shock protein, mitochondrial	HSP60	P10809	5	3.7	0.0006
19	Tyrosine-protein kinase Fyn	FYN	P06241	5	3.5	0.0004
20	Tyrosine-protein kinase Yes	YES1	P07947	5	3.5	0.0003
21	Creatine kinase M-type	CKM	P06732	2	3.5	0.0011
22	Mitogen-activated protein kinase 8	MAPK8	P45983	5	3.4	0.0007
23	L-lactate dehydrogenase B chain	LDHB	P07195	0.5	3.3	0.0005
24	40S ribosomal protein S3	RPS3	P23396	5	3.2	0.0005
25	PIK3CA/PIK3R1	PIK3CA PIK3R1	P42336	5	3.1	0.0001
26	40S ribosomal protein S3a	RPS3A	P61247	5	3.1	0.0006
27	Cytochrome c	CYCS	P99999	5	3.0	0.0013
28	Proto-oncogene tyrosine-protein kinase Src	SRC	P12931	5	3.0	0.0007
29	Protein kinase C alpha type	PRKCA	P17252	5	3.0	0.0006
30	D-dimer	FGA FGB FGG	P02671	0.5	2.9	0.0001
31	Calpain I	CAPN1 CAPNS1	P07384	2	2.9	0.0001
32	RAC-beta serine/threonine-protein kinase	AKT2	P31751	5	2.8	0.0010
33	Mitogen-activated protein kinase 14	MAPK14	Q16539	2	2.8	0.0005
34	Peptidyl-prolyl cis-trans isomerase D	PPID	Q08752	5	2.8	0.0008
35	Troponin I, cardiac muscle	TNNI3	P19429	5	2.8	0.0036
36	RAC-alpha/beta/gamma serine/threonine-protein kinase	AKT1 AKT2 AKT3	P31749	2	2.7	0.0007
37	Coiled-coil domain-containing protein 80	CCDC80	Q76M96	2	2.7	0.0002
38	Tyrosine-protein phosphatase non-receptor type 11	PTPN11	Q06124	5	2.6	0.0010
39	40S ribosomal protein S7	RPS7	P62081	5	2.5	0.0009
40	Heat shock protein HSP 90-alpha/beta	HSP90AA1/AB1	P07900	2	2.5	0.0004
41	Ribosomal protein S6 kinase alpha-3	RPS6KA3	P51812	5	2.5	0.0005
42	cAMP-dependent protein kinase catalytic subunit alpha	PRKACA	P17612	5	2.5	0.0012
43	Dual specificity mitogen-activated protein kinase kinase 1	MAP2K1	Q02750	5	2.4	0.0001
44	Dual specificity protein phosphatase 3	DUSP3	P51452	5	2.4	0.0020
45	Pro-opiomelanocortin	POMC	P01189	5	2.4	0.0006
46	Heat shock 70 kDa protein 1A	HSPA1A	P0DMV8	5	2.4	0.0004
47	Casein kinase II 2-alpha':2-beta heterotetramer	CSNK2A2 CSNK2B	P19784	5	2.4	0.0043
48	Glycogen synthase kinase-3 alpha/beta	GSK3A GSK3B	P49840	5	2.4	0.0011
49	Mitogen-activated protein kinase 12	MAPK12	P53778	5	2.4	0.0004
50	3-phosphoinositide-dependent protein kinase 1	PDPK1	O15530	5	2.3	0.0020
51	Heat shock cognate 71 kDa protein	HSPA8	P11142	2	2.3	0.0003
52	ATP-dependent RNA helicase DDX19B	DDX19B	Q9UMR2	5	2.3	0.0006
53	14-3-3 protein family	YWHAB	P31946	2	2.3	0.0003
54	Protein FAM3D	FAM3D	Q96BQ1	5	2.2	0.0001
55	NAD-dependent protein deacetylase sirtuin-2	SIRT2	Q8IXJ6	5	2.2	0.0014
56	Xaa-Pro aminopeptidase 1	XPNPEP1	Q9NQW7	5	2.2	0.0008
57	Receptor-type tyrosine-protein kinase FLT3	FLT3	P36888	5	2.2	0.0018
58	Protein kinase C beta type (splice variant beta-II)	PRKCB	P05771	2	2.2	0.0016
59	Tumor necrosis factor receptor superfamily member 27	EDA2R	Q9HAV5	5	2.2	0.0001
60	Osteocalcin	BGLAP	P02818	2	2.2	0.0012
61	Histone H2A.z	H2AFZ	P0C0S5	5	2.2	0.0001
62	Tyrosine-protein kinase CSK	CSK	P41240	2	2.1	0.0027
63	Mothers against decapentaplegic homolog 3	SMAD3	P84022	5	2.1	0.0016
64	Secreted and transmembrane protein 1	SECTM1	Q8WVN6	2	2.1	0.0003
65	Follistatin-related protein 1	FSTL1	Q12841	2	2.1	0.0004
66	beta-adrenergic receptor kinase 1	ADRBK1	P25098	5	2.1	0.0012
67	Hemojuvelin	HFE2	Q6ZVN8	2	2.0	0.0010
68	Intercellular adhesion molecule 2	ICAM2	P13598	2	2.0	0.0001
69	Alpha-soluble NSF attachment protein	NAPA	P54920	2	2.0	0.0006
70	Protein S100-A12	S100A12	P80511	2	2.0	0.0013
71	MAP kinase-activated protein kinase 3	MAPKAPK3	Q16644	2	2.0	0.0031
72	Repulsive guidance molecule A	RGMA	Q96B86	2	2.0	0.0005
73	Mitochondrial import inner membrane translocase subunit TIM14	DNAJC19	Q96DA6	5	2.0	0.0001
74	Tyrosine-protein kinase Fer	FER	P16591	5	2.0	0.0012
75	Triosephosphate isomerase	TPI1	P60174	2	2.0	0.0034
76	Growth factor receptor-bound protein 2	GRB2	P62993	5	2.0	0.0008
77	Hsp90 co-chaperone Cdc37	CDC37	Q16543	5	2.0	0.0012
78	Methionine aminopeptidase 2	METAP2	P50579	5	2.0	0.0010
79	Nidogen-1	NID1	P14543	2	2.0	0.0003
80	Contactin-1	CNTN1	Q12860	0.5	0.7	0.0013
81	Growth/differentiation factor 8	MSTN	O14793	0.5	0.7	0.0033
82	Tumor necrosis factor receptor superfamily member 25	TNFRSF25	Q93038	5	0.7	0.005
83	Desmoglein-2	DSG2	Q14126	0.5	0.4	0.0005

Table S1: Serum *mdx* protein biomarkers identified by SOMAscan.

Rank	Protein	Target	Uniprot	Dilution	Fold change (mdx vs C57)	pValue	Recovery score (Fiona)
1	Tumor necrosis factor receptor superfamily member 18	TNFRSF18/GITR	Q9Y5U5	5	25.4	0.0001	+++
2	Troponin I, fast skeletal muscle	TNNI2	P48788	5	10.2	0.0002	++
3	Myosin-binding protein C, slow-type	MYBPC1	Q00872	5	7.7	0.0003	++
4	Fibrinogen	FGA	P02671	0.5	6.5	0.0001	-
5	Calcium/calmodulin-dependent protein kinase type II subunit beta	CAMK2B	Q13554	5	5.9	0.0001	++
6	Fibrinogen gamma chain	FGG	P02679	0.5	4.8	0.0001	-
7	Adenylate kinase isoenzyme 1	AK1	P00568	2	4.7	0.0007	++
8	Acidic leucine-rich nuclear phosphoprotein 32 family member B	ANP32B	Q92688	2	4.6	0.0003	+++
9	Troponin T, cardiac muscle	TNNT2	P45379	5	4.6	0.0011	+++
10	Thrombospondin-4	THBS4	P35443	0.5	4.5	0.0002	+++
11	Calcium/calmodulin-dependent protein kinase type II subunit delta	CAMK2D	Q13557	5	4.5	0.0001	++
12	Serine protease HTRA2, mitochondrial	HTRA2	O43464	5	4.2	0.0001	+++
13	Calcium/calmodulin-dependent protein kinase type II subunit alpha	CAMK2A	Q9UQM7	5	4.2	0.0003	+++
14	Tyrosine-protein kinase Lyn	LYN	P07948	5	4.0	0.0003	++
15	Tyrosine-protein kinase Lyn, isoform B	LYN	P07948	5	4.0	0.0003	+++
16	Proliferating cell nuclear antigen	PCNA	P12004	5	4.0	0.0012	+++
17	Eukaryotic translation initiation factor 4 gamma 2	EIF4G2	P78344	5	4.0	0.0012	-
18	60 kDa heat shock protein, mitochondrial	HSP60	P10809	5	3.7	0.0006	+++
19	Tyrosine-protein kinase Fyn	FYN	P06241	5	3.5	0.0004	+++
20	Myoglobin	MB	P02144	2	3.5	0.0011	+++
21	Tyrosine-protein kinase Yes	YES1	P07947	5	3.5	0.0003	++
22	Creatine kinase M-type	CKM	P06732	2	3.5	0.0011	+
23	Mitogen-activated protein kinase 8	MAPK8	P45983	5	3.4	0.0007	+
24	L-lactate dehydrogenase B chain	LDHB	P07195	0.5	3.3	0.0005	++
25	40S ribosomal protein S3	RPS3	P23396	5	3.2	0.0005	-
26	PIK3CA/PIK3R1	PIK3CA PIK3R1	P42336	5	3.1	0.0001	++
27	40S ribosomal protein S3a	RPS3A	P61247	5	3.1	0.0006	+++
28	Cytochrome c	CYCS	P99999	5	3.0	0.0013	+++
29	Proto-oncogene tyrosine-protein kinase Src	SRC	P12931	5	3.0	0.0007	++
30	D-dimer	FGA FGB FGG	P02671	0.5	2.9	0.0001	-
31	Calpain I	CAPN1 CAPNS1	P07384	2	2.9	0.0001	+++
32	RAC-beta serine/threonine-protein kinase	AKT2	P31751	5	2.8	0.0010	-
33	Mitogen-activated protein kinase 14	MAPK14	Q16539	2	2.8	0.0005	+
34	Peptidyl-prolyl cis-trans isomerase D	PPID	Q08752	5	2.8	0.0008	-
35	Troponin I, cardiac muscle	TNNI3	P19429	5	2.8	0.0036	-
36	RAC-alpha/beta/gamma serine/threonine-protein kinase	AKT1 AKT2 AKT3	P31749	2	2.7	0.0007	-
37	Coiled-coil domain-containing protein 80	CCDC80	Q76M96	2	2.7	0.0002	++
38	Tyrosine-protein phosphatase non-receptor type 11	PTPN11	Q06124	5	2.6	0.0010	++
39	40S ribosomal protein S7	RPS7	P62081	5	2.5	0.0009	+++
40	Heat shock protein HSP 90-alpha/beta	HSP90AA1/AB1	P07900	2	2.5	0.0004	+
41	Ribosomal protein S6 kinase alpha-3	RPS6KA3	P51812	5	2.5	0.0005	+
42	cAMP-dependent protein kinase catalytic subunit alpha	PRKACA	P17612	5	2.5	0.0012	-
43	Dual specificity mitogen-activated protein kinase kinase 1	MAP2K1	Q02750	5	2.4	0.0001	-
44	Dual specificity protein phosphatase 3	DUS3	P51452	5	2.4	0.0020	+++
45	Heat shock 70 kDa protein 1A	HSPA1A	P0DMV8	5	2.4	0.0004	+
46	Casein kinase II 2-alpha':2-beta heterotetramer	CSNK2A2 CSNK2B	P19784	5	2.4	0.0043	+++
47	Glycogen synthase kinase-3 alpha/beta	GSK3A GSK3B	P49840	5	2.4	0.0011	-
48	Mitogen-activated protein kinase 12	MAPK12	P53778	5	2.4	0.0004	++
49	3-phosphoinositide-dependent protein kinase 1	PDPK1	O15530	5	2.3	0.0020	++
50	Heat shock cognate 71 kDa protein	HSPA8	P11142	2	2.3	0.0003	++
51	ATP-dependent RNA helicase DDX19B	DDX19B	Q9UMR2	5	2.3	0.0006	+
52	14-3-3 protein family	YWHAB	P31946	2	2.3	0.0003	++
53	Protein FAM3D	FAM3D	Q96BQ1	5	2.2	0.0001	+++
54	NAD-dependent protein deacetylase sirtuin-2	SIRT2	Q8IXJ6	5	2.2	0.0014	+++
55	Xaa-Pro aminopeptidase 1	XPNPEP1	Q9NQW7	5	2.2	0.0008	+++
56	Receptor-type tyrosine-protein kinase FLT3	FLT3	P36888	5	2.2	0.0018	+
57	Protein kinase C beta type (splice variant beta-II)	PRKCB	P05771	2	2.2	0.0016	-
58	Tumor necrosis factor receptor superfamily member 27	EDA2R	Q9HAV5	5	2.2	0.0001	+++
59	Osteocalcin	BGLAP	P02818	2	2.2	0.0012	+++
60	Histone H2A.z	H2AFZ	P0C0S5	5	2.2	0.0001	+++
61	Tyrosine-protein kinase CSK	CSK	P41240	2	2.1	0.0027	-
62	Mothers against decapentaplegic homolog 3	SMAD3	P84022	5	2.1	0.0016	+++
63	Secreted and transmembrane protein 1	SECTM1	Q8WVN6	2	2.1	0.0003	+++
64	Follistatin-related protein 1	FSTL1	Q12841	2	2.1	0.0004	+++
65	beta-adrenergic receptor kinase 1	ADRBK1	P25098	5	2.1	0.0012	+++
66	Hemojuvelin	HFE2	Q6ZVN8	2	2.0	0.0010	+++
67	Intercellular adhesion molecule 2	ICAM2	P13598	2	2.0	0.0001	+++
68	Alpha-soluble NSF attachment protein	NAPA	P54920	2	2.0	0.0006	+
69	Protein S100-A12	S100A12	P80511	2	2.0	0.0013	+++
70	MAP kinase-activated protein kinase 3	MAPKAPK3	Q16644	2	2.0	0.0031	-
71	Repulsive guidance molecule A	RGMA	Q96B86	2	2.0	0.0005	+++
72	Mitochondrial import inner membrane translocase subunit TIM14	DNAJC19	Q96DA6	5	2.0	0.0001	+++
73	Triosephosphate isomerase	TPI1	P60174	2	2.0	0.0034	++
74	Growth factor receptor-bound protein 2	GRB2	P62993	5	2.0	0.0008	-
75	Casein kinase II 2-alpha:2-beta heterotetramer	CSNK2A1 CSNK2B	P68400 P67870	5	2.0	0.0004	+++
76	Hsp90 co-chaperone Cdc37	CDC37	Q16543	5	2.0	0.0012	-
77	Methionine aminopeptidase 2	METAP2	P50579	5	2.0	0.001	+++
78	Nidogen-1	NID1	P14543	2	2.0	0.0003	+
79	Contactin-1	CNTN1	Q12860	0.5	0.7	0.0013	+++
80	Growth/differentiation factor 11/8	GDF11, MSTN	O95390 O14793	2	0.7	0.0036	+++
81	Bcl-2-like protein 2	BCL2L2	Q92843	5	0.7	0.0006	-
82	beta-nerve growth factor	NGF	P01138	5	0.7	0.0006	-
83	E3 ubiquitin-protein ligase ZNRF3	ZNRF3	Q9ULT6	5	0.7	0.0009	-
84	Growth/differentiation factor 8	MSTN	O14793	0.5	0.7	0.0033	+++
85	Tumor necrosis factor receptor superfamily member 25	TNFRSF25	Q93038	5	0.7	0.005	-
86	Neurogenic locus notch homolog protein 2	NOTCH2	Q04721	5	0.6	0.0005	-
87	Glucose-6-phosphate isomerase	GPI	P06744	5	0.6	0.0018	-
88	Plasminogen	PLG	P00747	0.5	0.4	0.0012	-
89	Desmoglein-2	DSG2	Q14126	0.5	0.4	0.0005	++

Table S2: Serum *mdx* protein biomarkers and rescue in *mdx* transgenic Fiona mice.

Target	Catalog Number	Detection range	Sensitivity	Comments
ANP32B	ABIN1745100		0.1ng/ml	
CAMK2A	ABIN426487	0.31-20 ng/mL	0.118 ng/mL	Sample below detection limit
GITR	ABIN1672796	62.5-4000 pg/mL	<10pg/mL	
SIRT2	ABIN416762	1.56-100 ng/mL	0.65 ng/mL	
THBS4	ABIN426909	0.312-20 ng/mL	0.64 ng/mL	
TNNT2	ABIN426397	15.63-1000 pg/mL	6.2 pg/mL	

Table S3: Details of the ELISA assays used in the study.

References

1 Schindelin, J. *et al.* Fiji: an open-source platform for biological-image analysis. *Nature methods* **9**, 676-682, doi:10.1038/nmeth.2019 (2012).

2 Barclay, C. J., Woledge, R. C. & Curtin, N. A. Effects of UCP3 genotype, temperature and muscle type on energy turnover of resting mouse skeletal muscle. *Pflugers Archiv : European journal of physiology* **457**, 857-864, doi:10.1007/s00424-008-0552-z (2009).

3 Lynch, G. S., Hinkle, R. T. & Faulkner, J. A. Power output of fast and slow skeletal muscles of mdx (dystrophic) and control mice after clenbuterol treatment. *Experimental physiology* **85**, 295-299 (2000).

4 Brooks, S. V. & Faulkner, J. A. Contractile properties of skeletal muscles from young, adult and aged mice. *The Journal of physiology* **404**, 71-82 (1988).
